# Establishing a novel Fanconi anemia signaling pathway-associated prognostic model and tumor clustering for pediatric acute myeloid leukemia patients

**DOI:** 10.1515/med-2023-0847

**Published:** 2023-11-09

**Authors:** Lixian Chang, Xuelian Cheng, Xingjie Gao, Yao Zou, Weiping Yuan, Li Zhang, Xiaofan Zhu

**Affiliations:** State Key Laboratory of Experimental Hematology, National Clinical Research Center for Blood Diseases, Haihe Laboratory of Cell Ecosystem, Institute of Hematology & Blood Diseases Hospital, Chinese Academy of Medical Sciences & Peking Union Medical College, Tianjin, 300020, China; Tianjin Institutes of Health Science, Tianjin, 301600, China; Department of Biochemistry and Molecular Biology, School of Basic Medical Science, Key Laboratory of Cellular and Molecular Immunology in Tianjin, The Province and Ministry Co-sponsored Collaborative Innovation Center for Medical Epigenetics, Tianjin Medical University, Tianjin, 300070, China; State Key Laboratory of Experimental Hematology, National Clinical Research Center for Blood Diseases, Haihe Laboratory of Cell Ecosystem, Institute of Hematology & Blood Diseases Hospital, Chinese Academy of Medical Sciences & Peking Union Medical College, Tianjin, 300020, China

**Keywords:** Fanconi anemia, acute myeloid leukemia, prognosis, children, expression

## Abstract

Considering the connection between the Fanconi anemia (FA) signaling pathway and tumor development, we aim to investigate the links between the FA gene expression and the survival prognosis of acute myeloid leukemia (AML) patients. Our study begins by identifying two distinct clusters of pediatric AML patients. Following the batch matching of the TARGET-AML, TCGA-LAML GSE71014, GSE12417, and GSE37642 cohorts, the samples were divided into a training set and an internal validation set. A Lasso regression modeling analysis was performed to identify five signatures: BRIP1, FANCC, FANCL, MAD2L2, and RFWD3. The AML samples were stratified into high- and low-risk groups by evaluating the risk scores. The AML high-risk patients showed a poorer overall survival prognosis. To predict the survival rates, we developed an FA Nomogram incorporating risk score, gender, age, and French–American–British classification. We further utilized the BEAT-AML cohort for the external validation of FA-associated prognostic models and observed good clinical validity. Additionally, we found a correlation between DNA repair, cell cycle, and peroxide-related metabolic events and FA-related high/low risk or cluster 1/2. In summary, our novel FA-associated prognostic models promise to enhance the prediction of pediatric AML prognosis.

## 1 Introduction

During myeloid differentiation, hematopoietic progenitor cells get blocked, resulting in acute myeloid leukemia (AML) [1,2,3]. AML is characterized by its high heterogeneity, associated with several factors, including cytogenetic abnormalities, genetic mutations, and changes in gene expression [1,2,3]. Pediatric AML constitutes approximately 15–20% of all cases of childhood leukemia [4,5]. Advancements in hematopoietic stem cell transplantation, molecular targeted therapy, immunotherapy, and genomic technology have improved prognosis and overall survival (OS) rates for pediatric AML. However, the mortality rate remains high [4,5]. Pediatric AML exhibits distinct genetic characteristics compared to adult AML [6]. Evaluating prognostic risk factors in AML is crucial for the management strategies or therapeutic advances in AML patients [1,2,3]. Several models have been reported for predicting the prognosis of AML patients, focusing on specific biological events [7,8,9]. For instance, a survival model based on 85 genes was reported to assess the AML prognosis [7]. Another study identified a 29-gene signature that could be a helpful predictor for therapy resistance in intensively treated adult AML patients [9]. However, fewer specific gene expression-based models or clusters were reported for predicting the prognosis of pediatric AML.

Fanconi anemia (FA) is characterized by autosomal or X-linked-recessive and autosomal-dominant genetic inheritance [10,11,12,13,14]. The pathogenesis of FA is associated with the FA signaling pathway, which contributes to genomic stability [10,11,12,13,14]. So far, 23 FA-related genes have been identified, such as FANCA/B/C and FANCD2 [10,11,12,13,14,15]. Extensive research has established strong connections between the FA signaling pathway and cancers [16,17,18,19]. FA patients are more susceptible to AML and other solid tumors [16,17,18,19,20,21]. For example, the FA pathway has shown potential as a treatment target for colorectal cancer [16]. Therefore, developing effective and reliable prognostic risk score models for AML is crucial by considering the expression pattern of FA signaling pathway genes.

Herein, we carried out a cluster analysis of pediatric AML cases based on the FA signaling pathway. We then identified five FA hub genes (including BRIP1, FANCC, FANCL, MAD2L2, and RFWD3). These genes were used to establish a novel FA-related prognostic model for pediatric AML patients.

## 2 Methods

### 2.1 Gene expression and clinical information

Gene expression matrix of the TPM (transcripts per million) type and a set of clinical traits were obtained from The Cancer Genome Atlas (TCGA)-LAML cohort using the “TCGAbiolinks” R package. We analyzed the expression pattern of each FA gene by combining datasets within a genotype-tissue expression (GTEx) and TCGA-LAML cohort. The data of TARGET-AML were obtained from the official website (https://ocg.cancer.gov/). We performed a statistical analysis using the wilcox.test or kruskal.test. The “GEOquery” R package was utilized to obtain the datasets of the GSE71014, GSE37642, and GSE12417 cohorts within the gene expression omnibus (GEO) database. Then, the expression matrix of TARGET-AML (*n* = 257), TCGA-LAML (*n* = 137), GSE71014 (*n* = 104), GSE37642 (*n* = 136), and GSE12417 (*n* = 78) was processed using the R language, and the batch correction was performed using the “sva” package. We then used the prcomp() function for the principal component analysis (PCA). The BEAT-AML cohort (*n* = 399) [22,23,24,25,26] was utilized as an external validation set.

### 2.2 Tumor clustering

We used the “ConsensusClusterPlus” R package [27] to cluster TARGET-AML samples according to the expression characteristics of FA signatures. Then, PCA was conducted. Additionally, we utilized the “survival” R package to perform the cluster-related prognostic analysis.

### 2.3 Lasso regression modeling construction

We performed Lasso regression modeling analysis using the datasets of TARGET-AML, TCGA-LAML GSE71014, GSE12417, and GSE37642 cohorts. The cases were divided into a training set (75%) and an internal validation set (25%). We used the “glmnet” R package to conduct a Lasso regression modeling analysis of the training set. The resulting model provided gene coefficients and risk score values. Risk scores were grouped according to their median values to determine high- and low-risk groups. The OS risk values for the internal validation set were also obtained. We plotted the risk profile, survival curves, and the survival status map. The hub gene expression profile was visualized as a heat map. Furthermore, we used the BEAT-AML cohort to externally validate the above FA-associated prognostic model.

### 2.4 Gene enrichment

Differentially expressed genes in two tumor clusters or risk groups were identified using a “limma” R package with a false discovery rate of 0.05 and a log2 fold change (log2FC) of 1. A “ggplot2” R package was used to generate an MA plot. Also, a heat map of FA genes and the clinical traits of age, gender, French–American–British (FAB), and clustering status were created. Gene enrichment analysis was performed, and the results were visualized using the “GOplot” R package [28]. Furthermore, gene set enrichment analysis (GSEA) was conducted by a “clusterProfiler” R package [29,30], and the results were visualized using the “gseaplot()” function of the enrichplot R package. Common members of differential genes between the risk and clustering groups were obtained, and the related gene ontology (GO)-Kyoto Encyclopedia of Genes and Genomes (KEGG) analyses were performed.

### 2.5 Cox regression

To assess the OS prognosis, the univariate/multivariate Cox regression analyses were conducted using a “survival” R package, considering the factors of prognostic risk score, FAB, gender, and age. Additionally, we performed external validation of the BEAT-AML cohort based on the multivariate Cox regression model associated with FA, using the predict() function. The forest plots were generated using the plot() R function. To evaluate the receiver operating characteristic (ROC) results for different survival times, we utilized the “survivalROC” R package and provided the value of area under the curve (AUC).

### 2.6 Nomogram prediction model

A cph () modeling analysis was performed using the “regplot” R package, and a Nomogram was obtained. The calibration curves of AML cases were plotted using the calibrate() function of the “ggstatsplot” R package. The C index value was obtained using the “rms” R package. In addition, the external validation dataset (BEAT-AML) was used to perform calibration curve analysis based on the FA Nomogram. The net reclassification improvement (NRI) and integrated discrimination improvement (IDI) analyses were conducted using the “survIDINRI” R package. Finally, the decision curve analysis (DCA) was performed using the “stdca.R” package from www.decisioncurveanalysis.org.

## 3 Results

### 3.1 Analytic strategy

**Figure 1:**
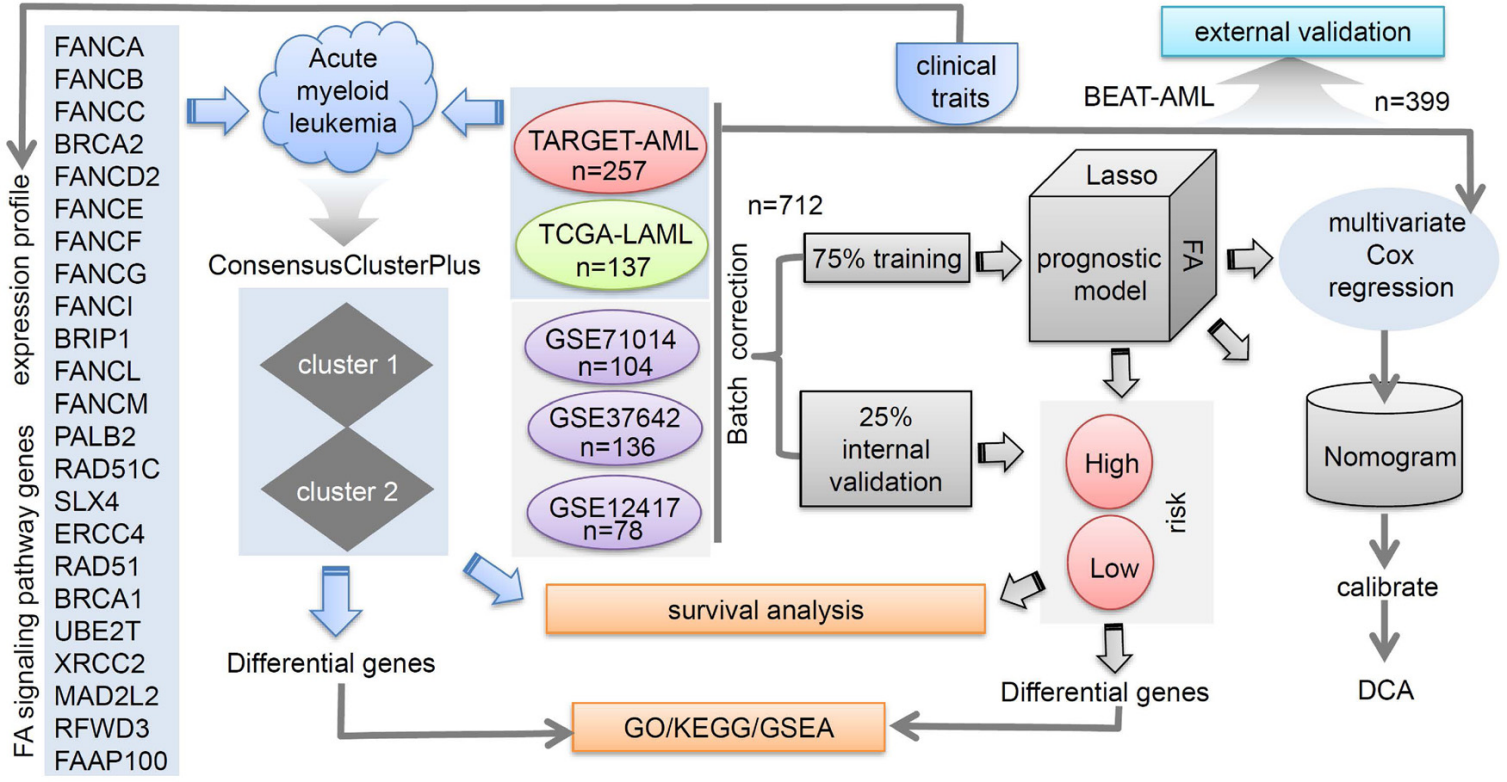
A schematic diagram of the study design.

The schematic diagram of our analytic strategy is presented in Figure 1. We initially analyzed the expression profile of 23 FA genes and the clinical traits. Next, using these FA signatures, we identified two tumor subtypes (cluster 1/2) in AML samples by utilizing the clustering function of the “ConsensusClusterPlus” R package. Additionally, we extracted and performed batch correction on the expression matrix of TARGET-AML, TCGA-LAML GSE71014, GSE12417, and GSE37642 cohorts. This was done with FA-related genes, the FA expression matrix, and corresponding clinical traits. An internal validation and training set were randomly selected from the samples. The training set-based Lasso regression modeling analysis was then conducted. We conducted multivariate Cox regression analyses. Nomograms and related calibrate curves were plotted as well. We assessed the clinical effectiveness of FA models by DCA. Additionally, survival, differential, and enrichment analyses of GO, KEGG, and GSEA were performed after risk or cluster grouping. FA-related models were validated in both the internal and external validation cohorts.

### 3.2 Expression profile

We analyzed the expression features of FA genes based on the combined datasets from the GTEx and TCGA-LAML cohorts.

**Figure 2:**
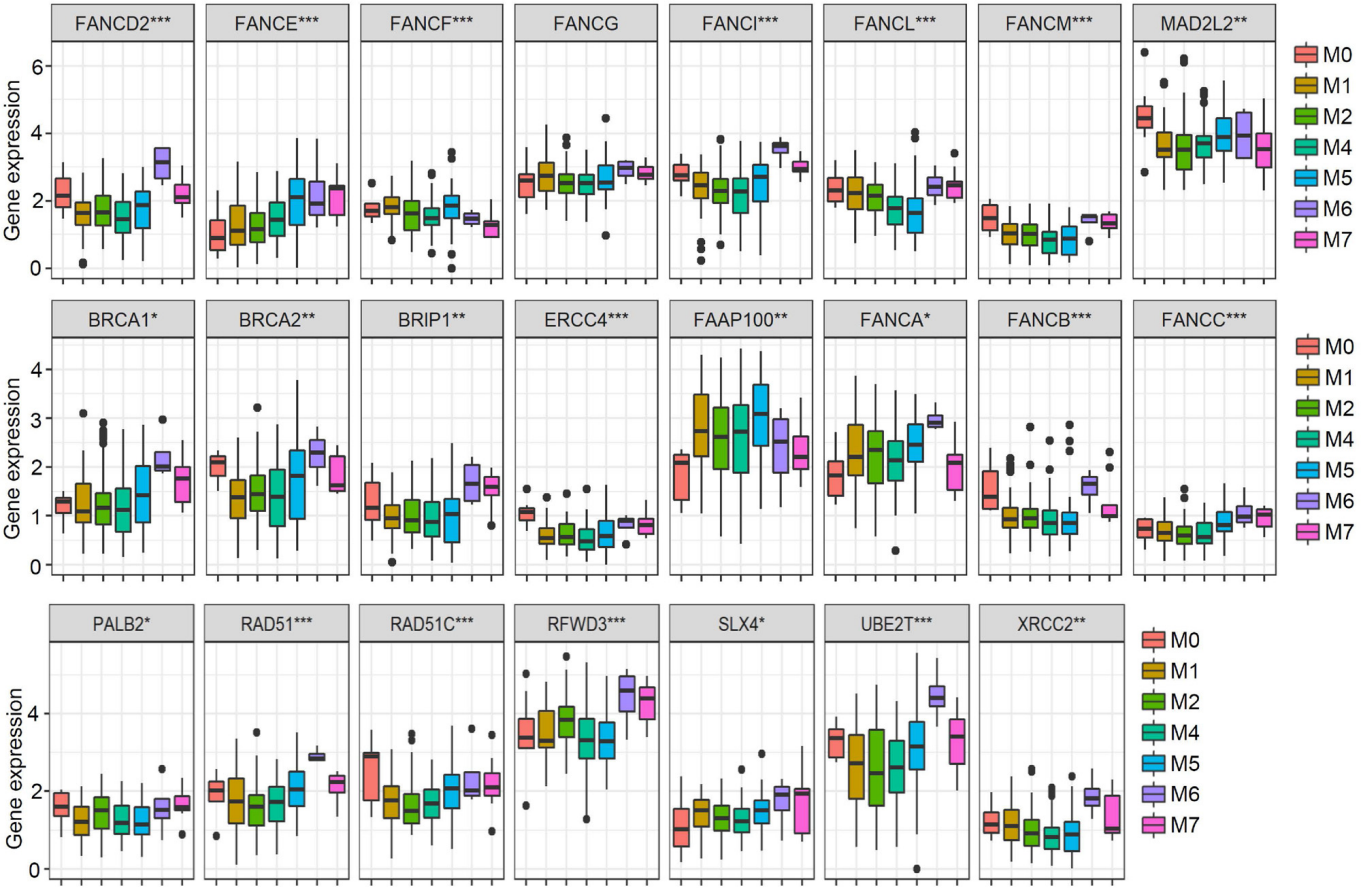
Correlation between FA gene expression and FAB. Box plots display each FA gene’s expression difference among different FAB types for the TARGET-AML cohort. A kruskal.test() was performed. *p < 0.05, **p < 0.01, ***p < 0.001.

As shown in Figure S1, we observed increased expression levels in the tumor group for FANCA, BRCA2, FANCG, FANCM, SLX4, ERCC4, and FAAP100 (all *p* < 0.05), while a decreased level was observed for FANCB, FANCC, FANCD2, FANCE, FANCI, FANCL, RAD51C, RAD51, BRCA1, UBE2T, XRCC2, MAD2L2, and RFWD3 (all *p* < 0.05) compared with the normal group. Additionally, there were expression differences for all FA genes, except FANCG, among different FAB types (Figure 2, all *p* < 0.05). We found a slightly higher expression of RAD51 (Figure S2, *p* < 0.05) and UBE2T (*p* < 0.05) in the male group. Furthermore, as shown in Figure S3, we observed positive correlations between the age factor and expression levels of FANCC and FANCE (*p* < 0.001).

### 3.3 Tumor clustering analysis

**Figure 3:**
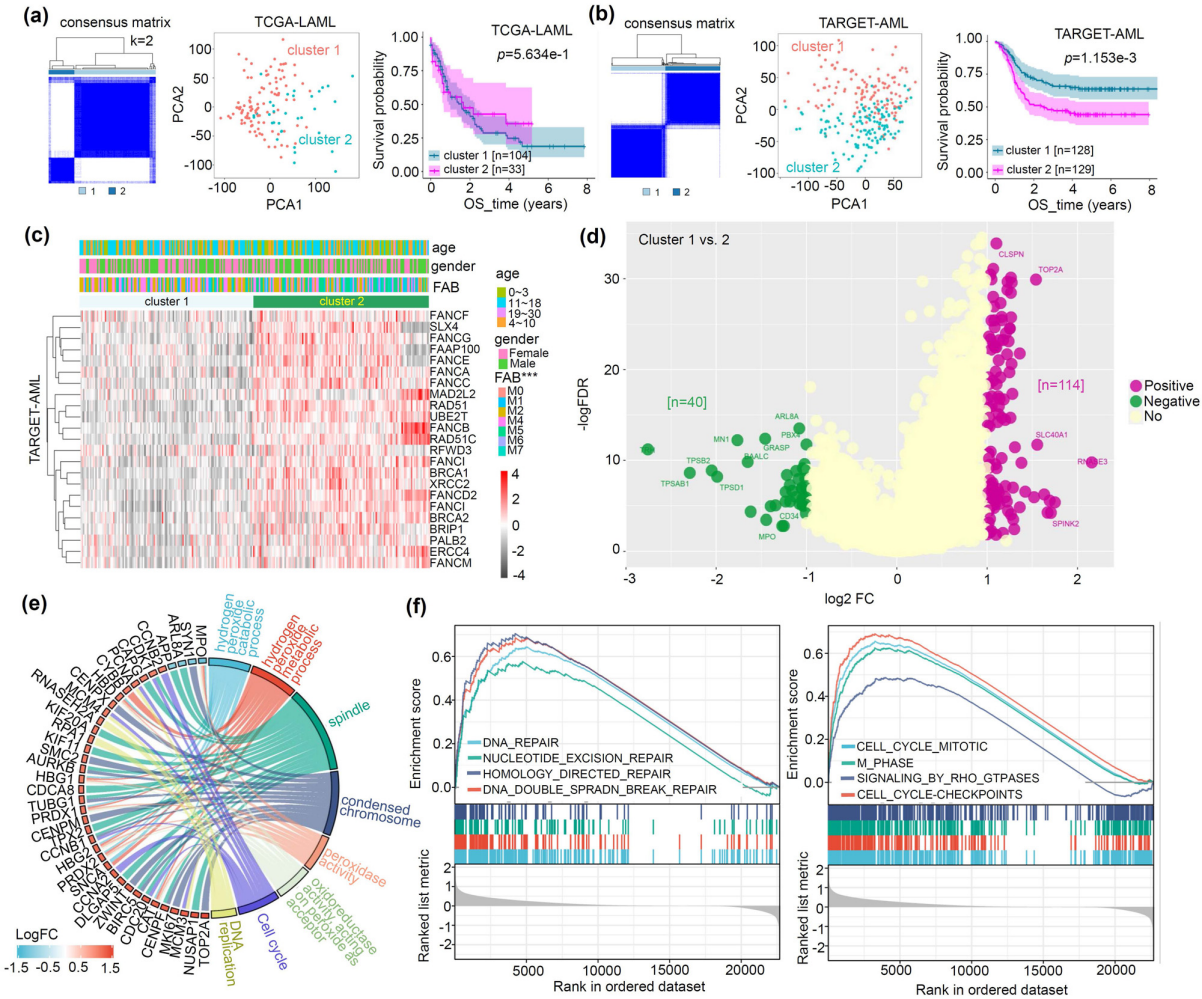
Tumor clustering analysis of FA genes. (a) AML patients from the TCGA-LAML were clustered according to the expression characteristics of FA-related genes. A PCA was performed to evaluate the classification effect on tumor clusters. The FA-specific prognostic curve of OS is also provided. (b) Similar analyses were conducted for the TARGET-AML cohort. (c) The correlation between FA gene expression and various clinical traits, including age, gender, FAB, and clusters, was analyzed. A heat map is provided. (d and e) The differential gene identification of the two clusters was completed, and a volcano plot is provided (d). The GO/KEGG (e) and GSEA (f) gene enrichment analyses were then conducted.

Next, we conducted a tumor clustering analysis based on the expression profile of FA genes within TCGA-LAML (Figure 3a) and TARGET-AML (Figure 3b) datasets. We separated these AML clusters using a PCA approach for both cohorts. However, we found a statistical difference in OS survival between the two clusters only for the TARGET-AML (Figure 3b, *p* = 1.153 × 10^−3^) but not for the TCGA-LAML (Figure 3a). Specifically, the pediatric AML patients of TARGET in cluster 2 showed a worse OS prognosis compared to those in cluster 1 (Figure 3b). We provided heat map data in Figure 3c, showing the association between FA gene expression and clinical information. Furthermore, a significant difference was observed in all FA gene expressions between clusters 1 and 2 (Figure S4, all *p* < 0.05).

The differential genes of the two clusters were obtained, and a volcano plot is shown in Figure 3d. The GO/KEGG enrichment analysis data (Figure 3e) revealed that these genes were associated with the events related to the “cell cycle,” “DNA replication,” and peroxide-related events such as “hydrogen peroxide metabolic process,” and “peroxidase activity.” The GSEA result further showed a series of genes linked to DNA repair events, such as “nucleotide excision repair” and “homology-directed repair,” as well as cell cycle events, such as “M_phase” and “cell cycle checkpoints” (Figure 3f). These findings suggest a connection between the pediatric AML FA subtypes and DNA repair, cell cycle, and peroxide-related metabolic events.

### 3.4 Lasso regression model

**Figure 4:**
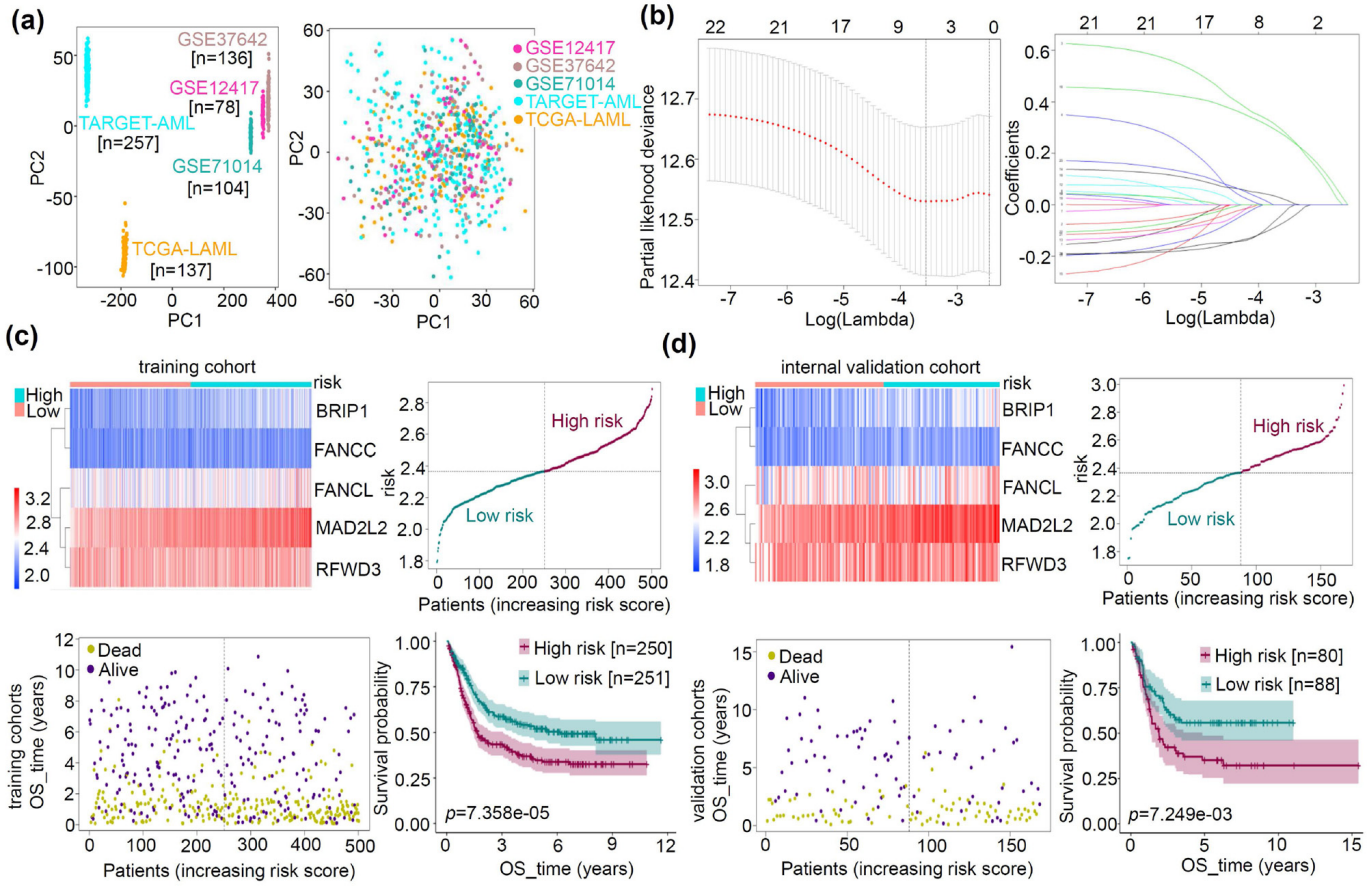
Lasso regression modeling analysis of FA. (a) We performed the Lasso regression modeling analysis based on the expression matrix of FA-related genes in TARGET-AML, TCGA-LAML GSE71014, GSE12417, and GSE37642 cohorts. A PCA plot shows the batch correction of the expression matrix. (b) Based on the training set, the Lasso regression modeling analysis was performed. (c and d) The data of the hub gene expression profile, risk profile, survival status map, and survival curve are provided in the training set (c) and internal validation set (d).

We initially conducted batch matching on the TARGET-AML, TCGA-LAML GSE71014, GSE12417, and GSE37642 cohorts (Figure 4a). Subsequently, we used 75% of the samples as a training set. Using the expression feature of FA genes, we performed a Lasso regression modeling analysis (Figure 4b). Our model identified five hub genes with correlation coefficients: BRIP1 (0.284), FANCC (−0.031), FANCL (0.022), MAD2L2 (0.270), and RFWD3 (−0.068). The results, including the expression/risk profile, survival curve, and survival status of the training and internal validation sets, are presented in Figure 4c–d. We observed an increased mortality status in AML cases as the risk value increased. Furthermore, the high-risk group exhibited a worse OS prognosis than the low-risk group (Figure 4c, *p* = 7.358 × 10^−5^; Figure 4d, *p* = 7.249 × 10^−3^).

### 3.5 Prognostic analysis

**Figure 5:**
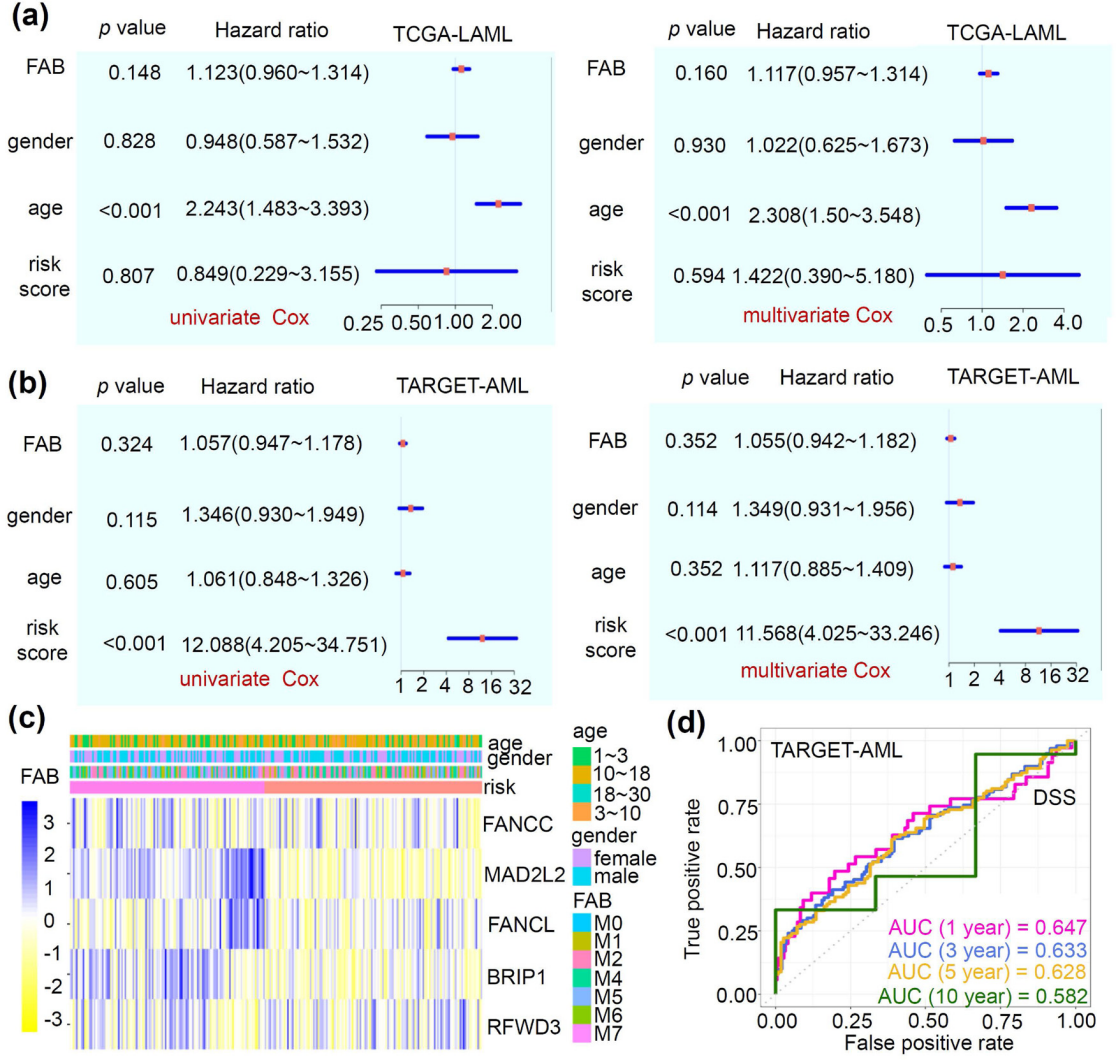
Prognostic analyses of TCGA-LAML and TARGET-AML. Targeting the factors (prognostic risk score, FAB, gender, and age), we performed the univariate/multivariate Cox regression analyses to evaluate the prognosis of OS for the TCGA-LAML (a) and TARGET-AML (b). We created a heatmap that combines the expression of hub genes with related clinical traits (c). Based on the risk score, we conducted the ROC analyses to assess the OS prognosis of TARGET-AML for different survival time points (d).

In the Cox regression analyses of TCGA-LAML, the age factor was found to be correlated with worse clinical OS prognosis in adult AML patients (Figure 5a, HR > 1, *p* < 0.001). For the pediatric AML cases in the TARGET-AML cohort, we observed a relationship between higher risk scores and worse clinical OS prognosis (Figure 5b, all HR > 10, *p* < 0.001). The relationship between hub gene expression and clinical traits was visualized as a heatmap in Figure 5c. Furthermore, we performed ROC analysis to assess the OS predictive value of our model. Figure 5d shows that the AUC values were greater than 0.62 for 1-, 3-, 5-year survival time. These suggested that our FA model exhibits good predictive value for OS in pediatric AML patients.

### 3.6 Nomogram

**Figure 6:**
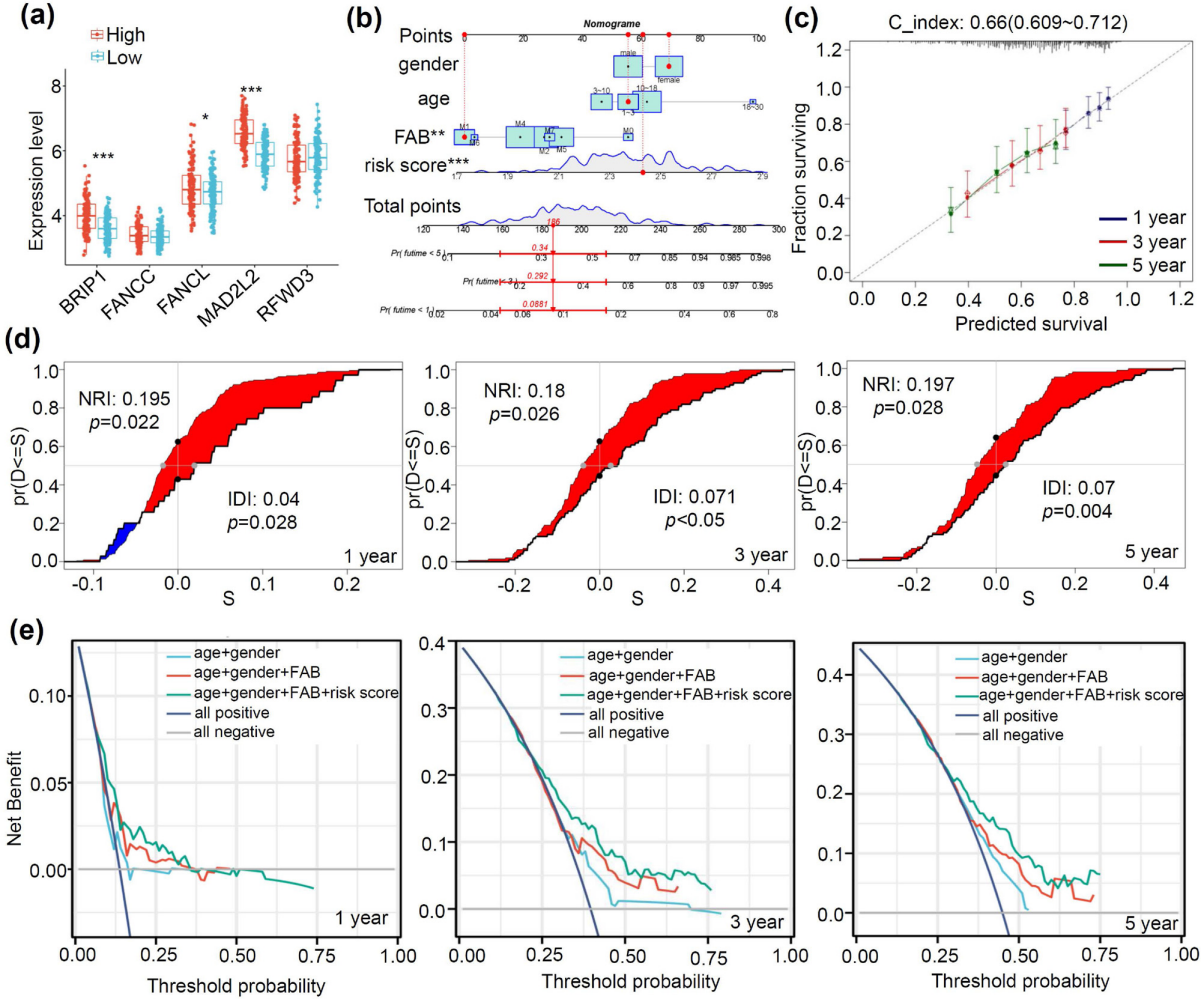
Nomogram and related assessment analyses. (a) The expression difference of FA hub genes between high- and low-risk groups is presented. (b) A Nomogram is constructed to forecast the 1-, 3-, and 5-year survival rates of a given AML case. Furthermore, the outcomes of the calibration plot curve (c), NRI/IDI assessment (d), and DCA (e) are provided.

We conducted the Nomogram and related assessments. Among the hub genes, there were higher expression levels of BRIP1 (Figure 6a, *p* < 0.001), FANCL (*p* < 0.05), and MAD2L2 (*p* < 0.001) in the high-risk group, compared with the low-risk group. By combining the risk score, gender, age, and FAB, we established a Nomogram to predict the OS rates of a pediatric AML case. The Nomogram, shown in Figure 6b, predicted the 1-, 3-, and 5-year survival rates at 0.34, 0.292, and 0.0881, respectively. The calibration plot curve in Figure 6c showed a high overlap between the predicted and observed value lines. The C index value of 0.66 was obtained (Figure 6c). Furthermore, NRI and IDI results suggested that the predictive function of the model improved after incorporating the risk score factor (Figure 6d, all IDI > 0, NRI > 0, *p* < 0.05). The DCA results (Figure 6e) further showed a better clinical validity of the “age + gender + FAB + risk score” group than other groups.

### 3.7 External validation analysis

**Figure 7:**
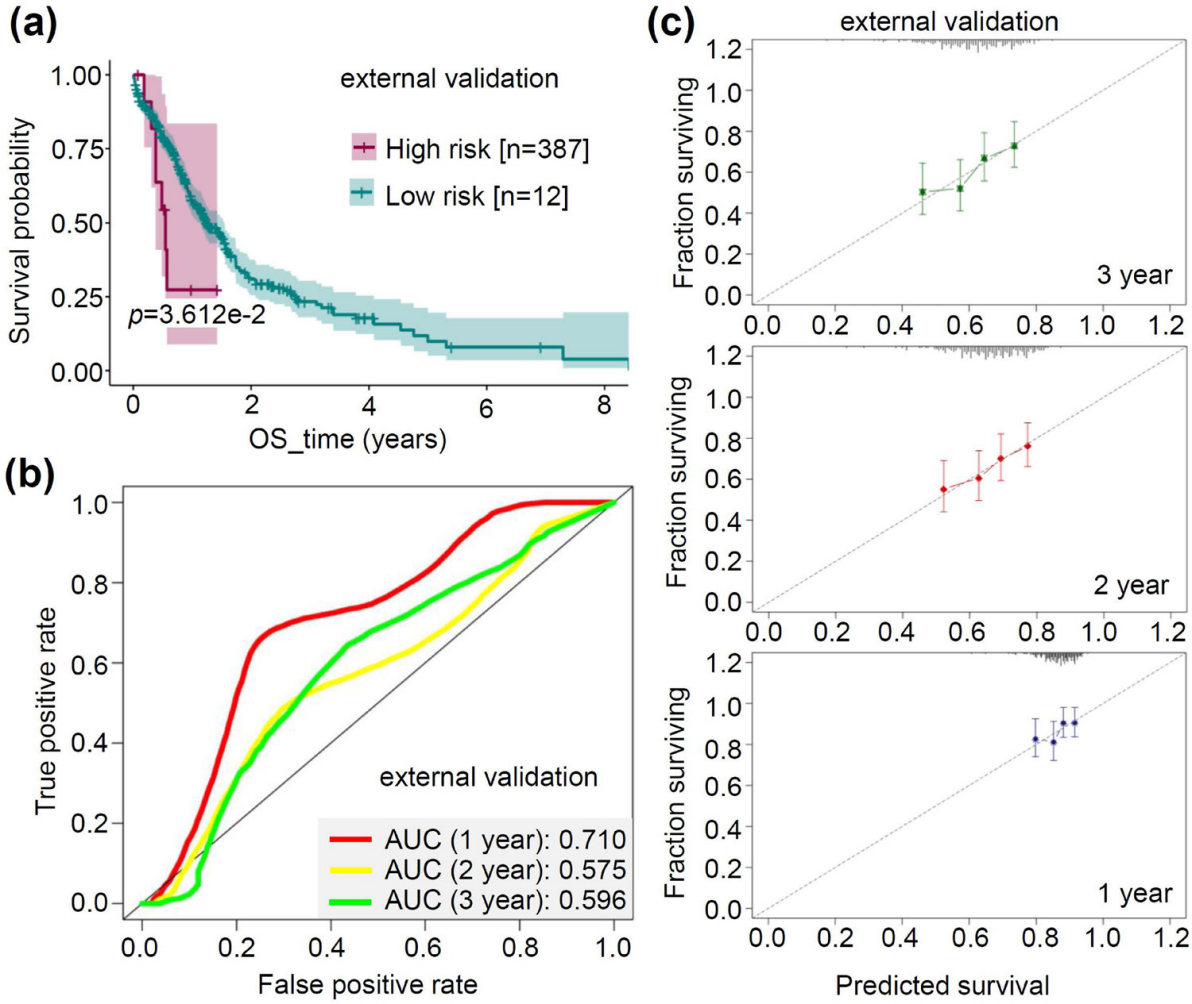
External validation of FA-associated prognostic models. (a) Based on the Lasso regression modeling of FA genes, we performed the survival curve analysis using the external validation cohorts (BEAT-AML). (b) The ROC results of the external validation set for 1-, 2-, and 3-year survival times were obtained based on the FA-associated multivariate Cox regression model. (c) Based on the FA Nomogram, we also performed the calibration curve analysis of the external validation set.

Based on the FA-associated prognostic models mentioned above, we conducted external validation using the BEAT-AML cohort. As shown in Figure 7a, it can be observed that AML cases with a high FA-associated risk score showed a poorer prognosis (*p* = 3.612 × 10^−2^). Furthermore, an external validation of the multivariate Cox regression model associated with FA was conducted, revealing a favorable predictive value for AML patients, particularly for the 1-year survival time (AUC = 0.71), as depicted in Figure 7b. A calibration plot curve was also generated using the external validation set, illustrating a substantial concurrence between the predicted and observed value lines (Figure 7c). These results serve to substantiate the good predictive capability of our FA-associated prognostic models.

### 3.8 Prognostic risk-related differential gene analysis

**Figure 8:**
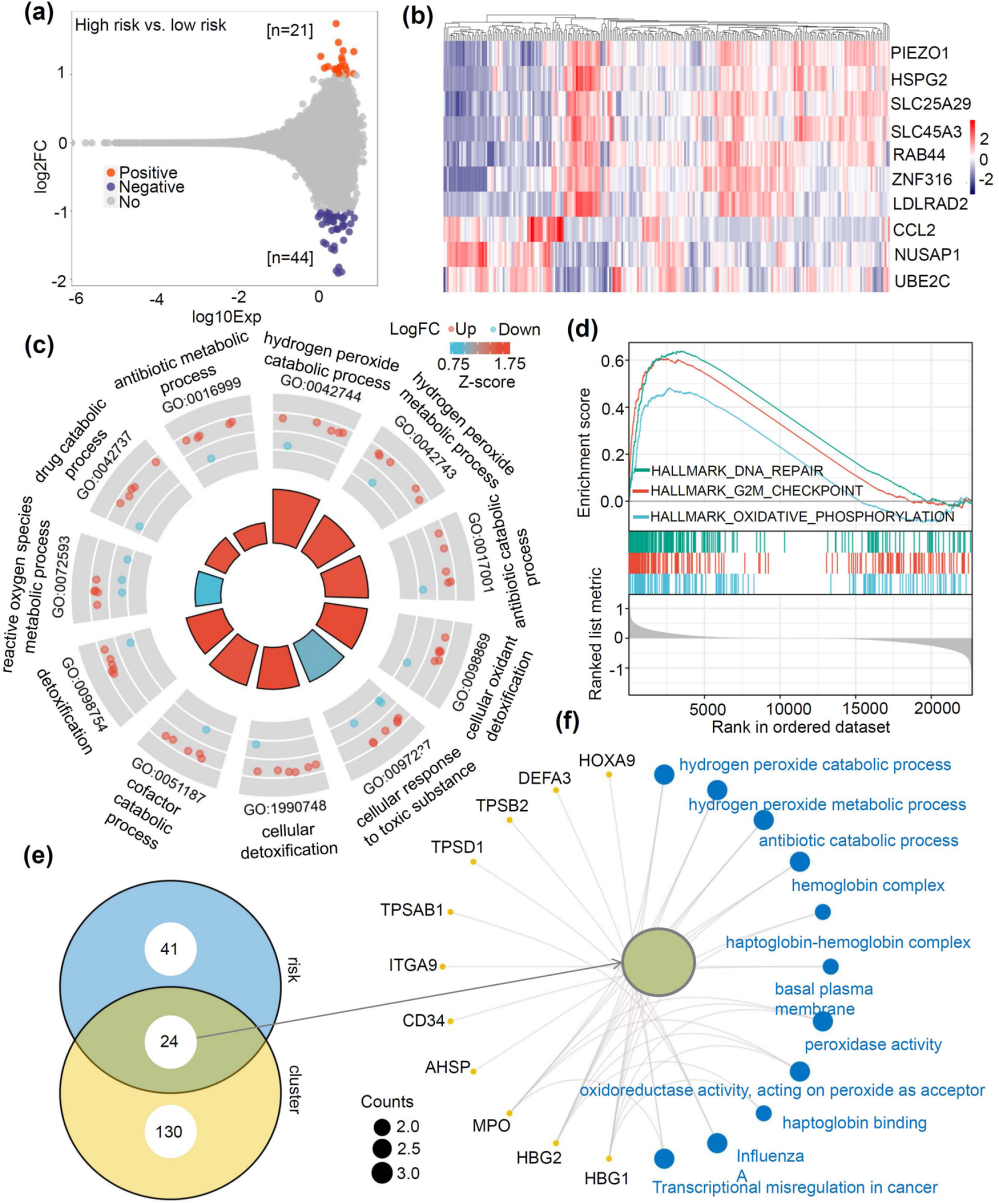
Prognostic risk-related differential gene analysis. (a and b) The differential gene identification between the high- and low-risk groups was performed, and the MA plot (a) and heat map (b) are provided to visualize the data. Gene enrichment analyses of GO-KEGG (c) and GSEA (d) were conducted to gain further insights. (e and f) Common members of differential genes between the risk and clustering groups were obtained, and gene enrichment analyses of GO-KEGG were performed to explore the functional significance.

We identified a series of differential genes (*n* = 65) based on the high- and low-risk grouping. The results were visualized via an MA plot (Figure 8a) and a heat map (Figure 8b). Further analysis of these differential genes was conducted using GO and KEGG enrichment approaches (Figure 8c). Our findings revealed the biological metabolic processes, such as the “antibiotic metabolic process” and “cellular oxidant detoxification.” Additionally, GSEA results showed the enrichment of events, including “DNA repair,” “G2M checkpoint,” and “oxidative phosphorylation” (Figure 8d). We also obtained 24 common members of differential genes between the risk and clustering groups (Figure 8e) and performed the gene enrichment analyses of GO-KEGG (Figure 8f). This analysis highlighted peroxide-related events, including the “hydrogen peroxide catabolic process” and “peroxidase activity.”

## 4 Discussion

We developed a prospective predictive model for AML by focusing on the FA signaling pathway. The consensus clustering of AML patients from the TCGA-LAML and TARGET-AML datasets was conducted using bioinformatics strategies. We identified two distinct clusters based on the expression patterns of FA signatures. Interestingly, we found a positive correlation between tumor clustering and OS prognosis only in pediatric AML cases but not in adult AML. Our novel FA-based tumor clustering approach may be helpful for the diagnosis and therapy of pediatric AML.

The close correlation between AML and mutations has led to the primary focus on correlation analysis of gene mutations in AML patients. This analysis aims to guide clinical treatment and evaluate prognosis [1,2,3,7,8,9,31]. For instance, in a previous study, we conducted a stratified prognostic analysis of different clinical subgroups in 132 children with AML-based data on gene mutations and related clinical traits [31]. Herein, we explore the potential correlation between FA-related models and the prognosis of AML patients, explicitly focusing on expression features of FA pathway genes. We developed a novel FA model for AML using the Lasso approach. Our multivariate Cox regression analysis also showed that a higher risk score is linked to a worse clinical OS prognosis, specifically in the TARGET-AML cohort rather than the TCGA-LAML cohort. These findings suggest a potential solid link between the FA signaling pathway and pediatric AML. Notably, the pediatric AML patients in cluster 2 or high-risk groups showed a worse survival prognosis.

In addition to conducting internal validation of models, we sought external validation of FA-related models by including independent external data. Due to the absence of high-throughput gene expression data for AML patients at our center, we utilized the BEAT AML data. It is worth noting that the BEAT-AML dataset had limited information on children’s AML. Thus, we refrained from filtering the BETA-AML data. The BEAT AML cohort was stratified into high- and low-risk groups based on our FA Lasso model, and the high-risk group exhibited a worse OS prognosis. Using FA-associated multivariate Cox regression analysis, we discovered that OS demonstrated favorable predictive capability for AML patients in the external validation cohort, particularly for the 1-year survival timeframe. However, we did not observe a statistically significant predictive effect for the 5-year survival period, potentially due to our analysis’s limited data on pediatric AML.

To enhance the predictive accuracy and clinical competence of survival prediction, we build an FA-associated Nomogram by combining the risk score, gender, age, and FAB. The calibration curve of both the training and external validation sets showed a significant overlap between the predicted and observed value lines, indicating strong predictive competence and accurate survival prediction. The findings of the FA model indicate that, along with mutation analysis of specific genes, it is crucial to measure the expression pattern of FA pathway genes in pediatric AML data. Standardization of FA expression detection procedures and continuous optimization of FA-related models will contribute to prognostic assessment and treatment decisions for children with AML. In addition, some patients with FA disease are known to develop tumors [21,32]. Previous studies have shown that Azacitidine effectively treats pediatric patients with FA and AML [20]. It is worth investigating the molecular mechanism underlying the transformation from FA to tumor and conducting specific chemosensitivity analysis. Once sufficient sample information is obtained, this can be achieved by studying the expression and mutation profile of FA-related genes.

The involvement of FA genes in DNA repair is closely linked to genomic maintenance within cells under stress conditions [10,33]. Despite being a rare chromosomal instability disorder [32], the functional loss of the FA signaling pathway during the DNA repair process is crucial for the occurrence and development of certain tumors [17,18,34]. Two components of the FA signaling pathway, namely BRCA1 and BRCA2, function as susceptibility genes for breast cancer [21]. Our study identified five hub prognostic genes in our model: BRIP1, FANCC, FANCL, MAD2L2, and RFWD3. As expected, clustering and risk-related gene enrichment analyses revealed a set of DNA repair-related events, including “nucleotide excision repair” and “homology-directed repair.” Besides, we observed enrichment of the cell cycle events, such as “M_phase,” “cell cycle checkpoints,” and “G2M checkpoint.” These findings suggest that the failure of timely and effective repair of damaged DNA during cell division is a critical factor in carcinogenesis when exposed to harmful external stimuli. Interestingly, we have seen the enrichment of peroxide-related biological metabolic issues, such as the “hydrogen peroxide catabolic process” and “peroxidase activity.” It has been observed that cells of individuals with FA are particularly vulnerable to oxidative stress, which is associated with the FANCG protein in mitochondria and peroxidase activity [35]. These findings suggest that the disruption in the peroxidase metabolic process contributes to the accumulation of oxidative DNA damage during tumorigenesis.

## 5 Conclusion

Overall, this study employed the expression data of FA-related genes to conduct a tumor clustering analysis. Also, a novel clinical predictive model was established, incorporating five FA-related signatures (BRIP1, FANCC, FANCL, MAD2L2, and RFWD3) for patients diagnosed with AML. Furthermore, a novel FA Nomogram was developed, demonstrating improved validity in predicting clinical survival outcomes. The FA-related prognostic model and tumor clustering have the potential to serve as prognostic predictors, aiding clinicians in evaluating the prognosis of pediatric AML patients.

## Abbreviations

AML: acute myeloid leukemia
FA: Fanconi anemia
FAB: French–American–British
OS: overall survival
TCGA: The Cancer Genome Atlas
GEO: gene expression omnibus
PCA: principal component analysis
TPM: transcripts per million
GTEx: genotype-tissue expression
FDR: false discovery rate
FC: fold change
GSEA: gene set enrichment analysis
GO: gene ontology
KEGG: Kyoto Encyclopedia of Genes and Genomes
ROC: receiver operating characteristic
AUC: area under the curve
NRI: net reclassification improvement
IDI: integrated discrimination improvement
DCA: decision curve analysis

## Supplementary Material

Supplementary Figure

## References

[1] Rubnitz JE, Kaspers GJL. How I treat pediatric acute myeloid leukemia. Blood. 2021;138(12):1009–18. 10.1182/blood.2021011694 34115839

[2] Short NJ, Rytting ME, Cortes JE. Acute myeloid leukaemia. Lancet. 2018;392(10147):593–606. 10.1016/S0140-6736(18)31041-9 PMC10230947 30078459

[3] Newell LF, Cook RJ. Advances in acute myeloid leukemia. BMJ. 2021;375:n2026. 10.1136/bmj.n2026 34615640

[4] Obszański P, Kozłowska A, Wańcowiat J, Twardowska J, Lejman M, Zawitkowska J. Molecular-targeted therapy of pediatric acute myeloid leukemia. Molecules. 2022;27(12):3911. 10.3390/molecules27123911 PMC9230975 35745032

[5] Tosic N, Marjanovic I, Lazic J. Pediatric acute myeloid leukemia: Insight into genetic landscape and novel targeted approaches. Biochem Pharmacol. 2023;215:115705. 10.1016/j.bcp.2023.115705 37532055

[6] Shiba N. Comprehensive molecular understanding of pediatric acute myeloid leukemia. Int J Hematol. 2023;117(2):173–81. 10.1007/s12185-023-03533-x 36653696

[7] Lai Y, Sheng L, Wang J, Zhou M, OuYang G. A novel 85-gene expression signature predicts unfavorable prognosis in acute myeloid leukemia. Technol Cancer Res Treat. 2021;20:15330338211004933. 10.1177/15330338211004933 PMC8020099 33784904

[8] Chen Z, Song J, Wang W, Bai J, Zhang Y, Shi J, et al. A novel 4-mRNA signature predicts the overall survival in acute myeloid leukemia. Am J Hematol. 2021;96(11):1385–95. 10.1002/ajh.26309 34339537

[9] Herold T, Jurinovic V, Batcha AMN, Bamopoulos SA, Rothenberg-Thurley M, Ksienzyk B, et al. A 29-gene and cytogenetic score for the prediction of resistance to induction treatment in acute myeloid leukemia. Haematologica. 2018;103(3):456–65. 10.3324/haematol.2017.178442 PMC5830382 29242298

[10] Ceccaldi R, Sarangi P, D’Andrea AD. The Fanconi anaemia pathway: New players and new functions. Nat Rev Mol Cell Biol. 2016;17(6):337–49. 10.1038/nrm.2016.48 27145721

[11] Mamrak NE, Shimamura A, Howlett NG. Recent discoveries in the molecular pathogenesis of the inherited bone marrow failure syndrome Fanconi anemia. Blood Rev. 2017;31(3):93–9. 10.1016/j.blre.2016.10.002 PMC5391297 27760710

[12] Inano S, Sato K, Katsuki Y, Kobayashi W, Tanaka H, Nakajima K, et al. RFWD3-mediated ubiquitination promotes timely removal of both RPA and RAD51 from DNA damage sites to facilitate homologous recombination. Mol Cell. 2017;66(5):622–34.e8. 10.1016/j.molcel.2017.04.022 28575658

[13] Nepal M, Che R, Ma C, Zhang J, Fei P. FANCD2 and DNA damage. Int J Mol Sci. 2017;18(8):1804. 10.3390/ijms18081804 PMC5578191 28825622

[14] Knies K, Inano S, Ramírez MJ, Ishiai M, Surrallés J, Takata M, et al. Biallelic mutations in the ubiquitin ligase RFWD3 cause Fanconi anemia. J Clin Invest. 2017;127(8):3013–27. 10.1172/JCI92069 PMC5531404 28691929

[15] Landelouci K, Sinha S, Pepin G. Type-I interferon signaling in Fanconi anemia. Front Cell Infect Microbiol. 2022;12:820273. 10.3389/fcimb.2022.820273 PMC8859461 35198459

[16] Parsa FG, Nobili S, Karimpour M, Aghdaei HA, Nazemalhosseini-Mojarad E, Mini E. Fanconi anemia pathway in colorectal cancer: A novel opportunity for diagnosis, prognosis and therapy. J Pers Med. 2022;12(3):396. 10.3390/jpm12030396 PMC8950345 35330396

[17] Nepal M, Che R, Zhang J, Ma C, Fei P. Fanconi anemia signaling and cancer. Trends Cancer. 2017;3(12):840–56. 10.1016/j.trecan.2017.10.005 PMC5819365 29198440

[18] Nalepa G, Clapp DW. Fanconi anaemia and cancer: An intricate relationship. Nat Rev Cancer. 2018;18(3):168–85. 10.1038/nrc.2017.116 29376519

[19] Savage SA, Walsh MF. Myelodysplastic syndrome, acute myeloid leukemia, and cancer surveillance in Fanconi anemia. Hematol Oncol Clin North Am. 2018;32(4):657–68. 10.1016/j.hoc.2018.04.002 PMC6071325 30047418

[20] Ding H, Hashem H, Cabral L, Rangarajan H, Abusin G, Lazarus HM, et al. Azacitidine as a bridge to allogeneic hematopoietic cell transplantation in a pediatric patient with Fanconi anemia and acute myeloid leukemia. Pediatr Transpl. 2017;21(2):e12870. 10.1111/petr.12870 27976488

[21] D’Andrea AD. Susceptibility pathways in Fanconi’s anemia and breast cancer. N Engl J Med. 2010;362(20):1909–19. 10.1056/NEJMra0809889 PMC3069698 20484397

[22] Tyner JW, Tognon CE, Bottomly D, Wilmot B, Kurtz SE, Savage SL, et al. Functional genomic landscape of acute myeloid leukaemia. Nature. 2018;562(7728):526–31. 10.1038/s41586-018-0623-z PMC6280667 30333627

[23] Papaemmanuil E, Gerstung M, Bullinger L, Gaidzik VI, Paschka P, Roberts ND, et al. Genomic classification and prognosis in acute myeloid leukemia. N Engl J Med. 2016;374(23):2209–21. 10.1056/NEJMoa1516192 PMC4979995 27276561

[24] Papaemmanuil E, Gerstung M, Malcovati L, Tauro S, Gundem G, Van Loo P, et al. Clinical and biological implications of driver mutations in myelodysplastic syndromes. Blood. 2013;122(22):3616–27; quiz 99. 10.1182/blood-2013-08-518886 PMC3837510 24030381

[25] Cerami E, Gao J, Dogrusoz U, Gross BE, Sumer SO, Aksoy BA, et al. The cBio cancer genomics portal: an open platform for exploring multidimensional cancer genomics data. Cancer Discov. 2012;2(5):401–4. 10.1158/2159-8290.CD-12-0095 PMC3956037 22588877

[26] Gao J, Aksoy BA, Dogrusoz U, Dresdner G, Gross B, Sumer SO, et al. Integrative analysis of complex cancer genomics and clinical profiles using the cBioPortal. Sci Signal. 2013;6(269):pl1. 10.1126/scisignal.2004088 PMC4160307 23550210

[27] Wilkerson MD, Hayes DN. ConsensusClusterPlus: A class discovery tool with confidence assessments and item tracking. Bioinformatics. 2010;26(12):1572–3. 10.1093/bioinformatics/btq170 PMC2881355 20427518

[28] Walter W, Sánchez-Cabo F, Ricote M. GOplot: an R package for visually combining expression data with functional analysis. Bioinformatics. 2015;31(17):2912–4. 10.1093/bioinformatics/btv300 25964631

[29] Yu G, Wang LG, Han Y, He QY. clusterProfiler: An R package for comparing biological themes among gene clusters. Omics. 2012;16(5):284–7. 10.1089/omi.2011.0118 PMC3339379 22455463

[30] Subramanian A, Tamayo P, Mootha VK, Mukherjee S, Ebert BL, Gillette MA, et al. Gene set enrichment analysis: a knowledge-based approach for interpreting genome-wide expression profiles. Proc Natl Acad Sci U S A. 2005;102(43):15545–50. 10.1073/pnas.0506580102 PMC1239896 16199517

[31] Liu LP, Zhang AL, Ruan M, Chang LX, Liu F, Chen X, et al. Prognostic stratification of molecularly and clinically distinct subgroup in children with acute monocytic leukemia. Cancer Med. 2020;9(11):3647–55. 10.1002/cam4.3023 PMC7286455 32216042

[32] Gueiderikh A, Maczkowiak-Chartois F, Rosselli F. A new frontier in Fanconi anemia: From DNA repair to ribosome biogenesis. Blood Rev. 2022;52:100904. 10.1016/j.blre.2021.100904 34750031

[33] Longerich S, Li J, Xiong Y, Sung P, Kupfer GM. Stress and DNA repair biology of the Fanconi anemia pathway. Blood. 2014;124(18):2812–9. 10.1182/blood-2014-04-526293 PMC4314529 25237197

[34] Niedernhofer LJ, Lalai AS, Hoeijmakers JH. Fanconi anemia (cross)linked to DNA repair. Cell. 2005;123(7):1191–8. 10.1016/j.cell.2005.12.009 16377561

[35] Mukhopadhyay SS, Leung KS, Hicks MJ, Hastings PJ, Youssoufian H, Plon SE. Defective mitochondrial peroxiredoxin-3 results in sensitivity to oxidative stress in Fanconi anemia. J Cell Biol. 2006;175(2):225–35. 10.1083/jcb.200607061 PMC2064564 17060495

