# Supplementary material for: Establishing a novel Fanconi anemia signaling pathway-associated prognostic model and tumor clustering for pediatric acute myeloid leukemia patients
Source: Open Med (Wars). 2023 Nov 9;18(1):20230847. doi: 10.1515/med-2023-0847 (PMC10655686; doi:10.1515/med-2023-0847)
Supplement: Supplementary Figure [file med-2023-0847-sm.pdf]

Supplementary material

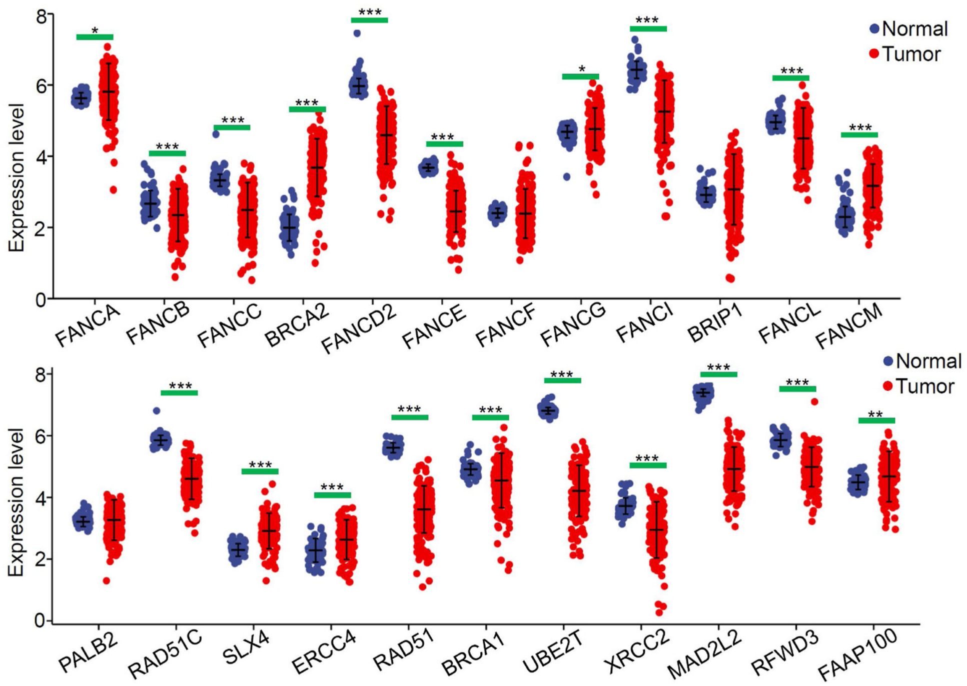

Figure S1: The expression difference between AML and controls for FA genes. Using the combined datasets within the GTEx and TCGA-LAML cohort, the expression difference of FA genes between the tumor and normal samples for FA genes was analyzed. A wilcox.test was conducted. * $p < 0.05$, ** $p < 0.01$, *** $p < 0.001$.

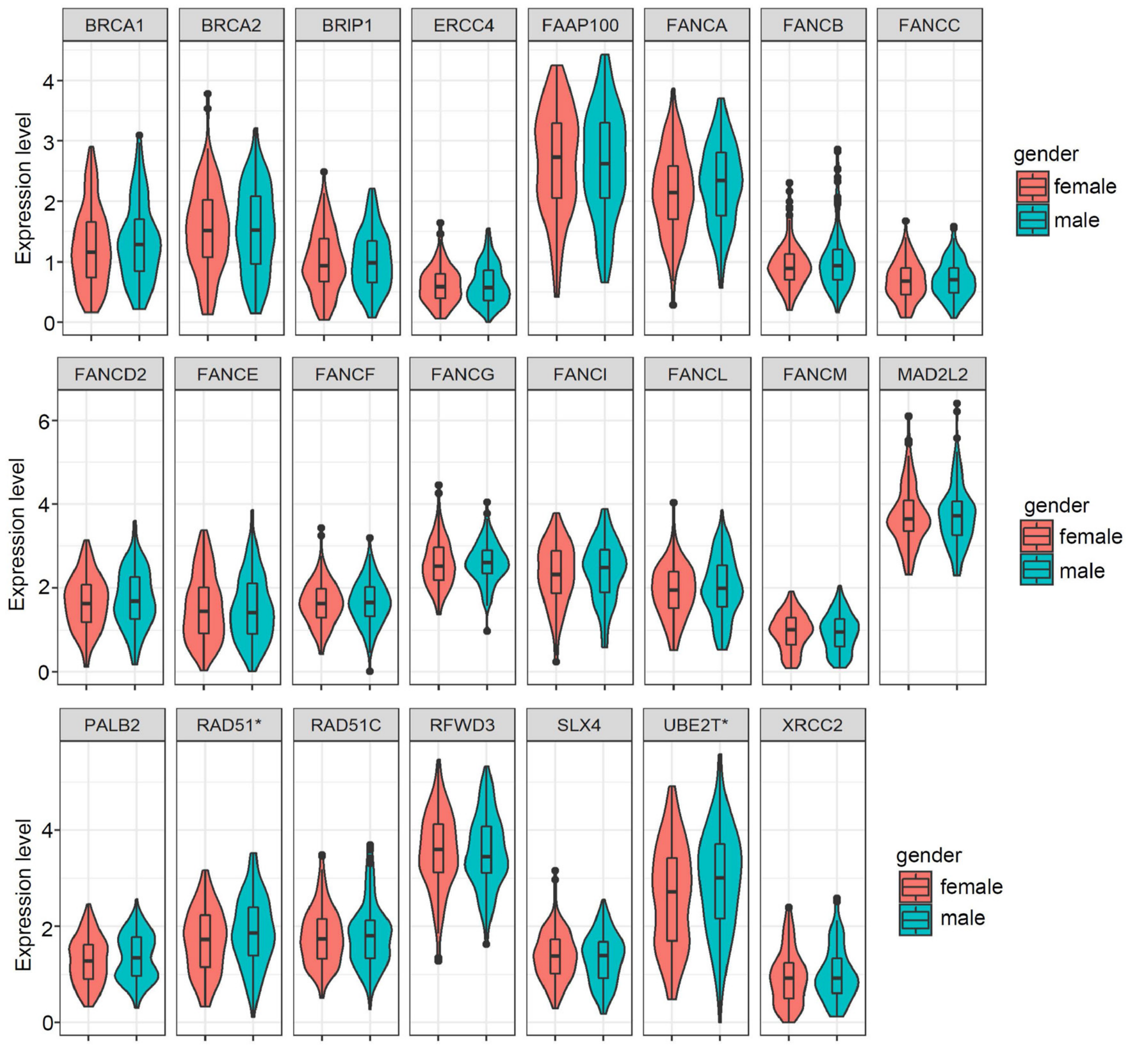

Figure S2: Correlation between FA gene expression and gender. Using data from the TARGET-AML cohort, we examined the expression differences of each FA gene between female and male groups. A wilcox.test was conducted. * $p < 0.05$.

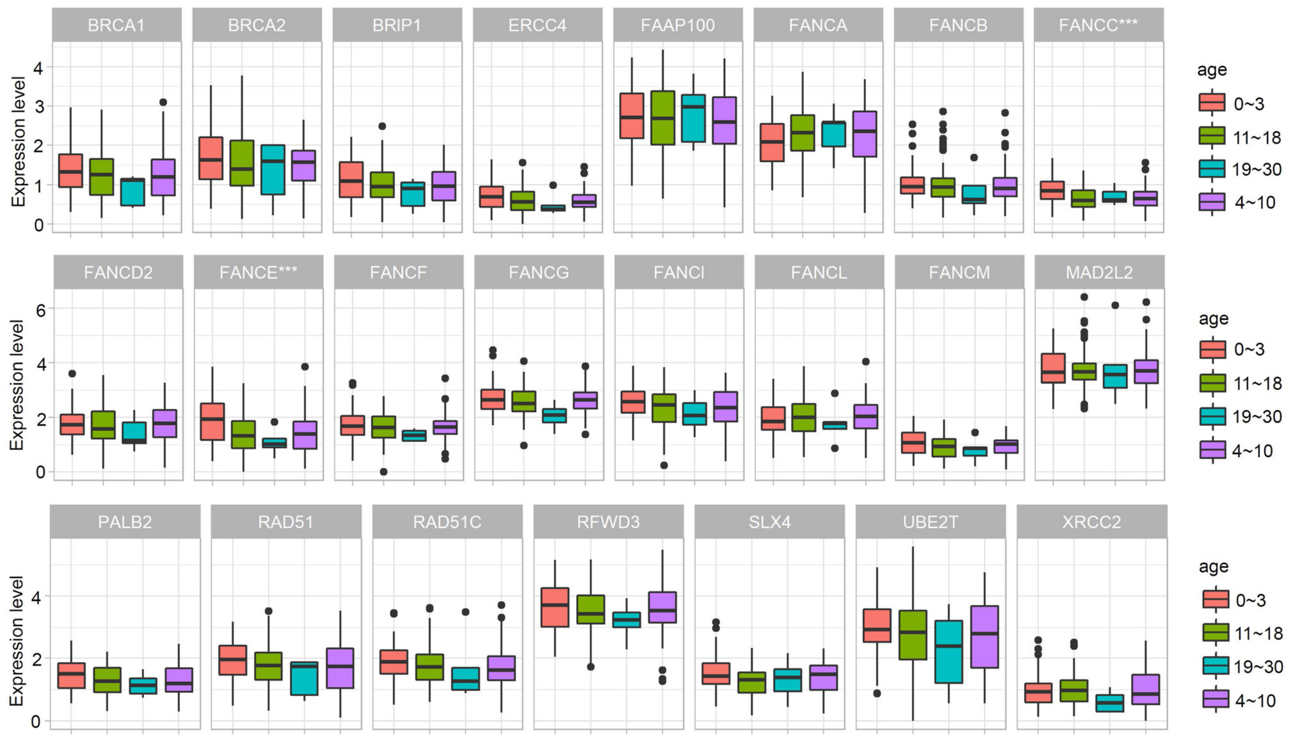

Figure S3: Correlation between FA gene expression and age. Using data from the TARGET-AML cohort, we investigated the variation in the expression of each FA gene across different age groups. Statistical analysis was conducted using a kruskal.test. *** $p < 0.001$.

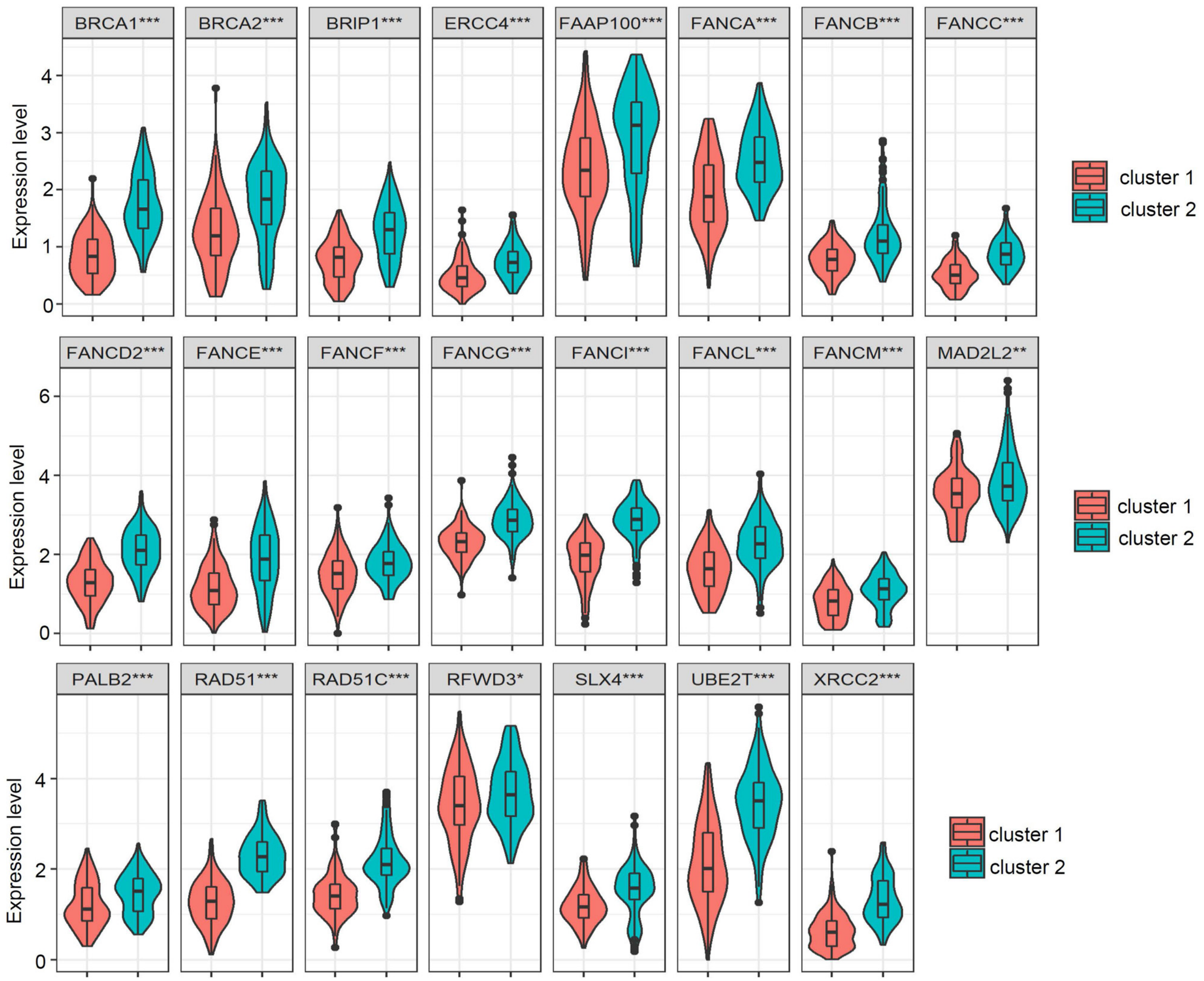

Figure S4: Expression difference of various FA genes between the two different clusters of the TARGET-AML cohort. A wilcox.test was conducted. * $p < 0.05$, ** $p < 0.01$, *** $p < 0.001$.
